# Intensity-modulated radiotherapy versus radical prostatectomy in patients with localized prostate cancer: long-term follow-up

**DOI:** 10.1186/1471-2407-13-530

**Published:** 2013-11-08

**Authors:** Tomás Merino, Ignacio F San Francisco, Pablo A Rojas, Piero Bettoli, Álvaro Zúñiga, Pelayo Besa

**Affiliations:** 1 Radiotherapy Unit, School of Medicine, Pontificia Universidad Católica de Chile, Marcoleta 352, Santiago, Chile; 2 Urology Department, School of Medicine, Pontificia Universidad Católica de Chile, Santiago, Chile; 3 Radiotherapy Unit, Clínica las Condes, Santiago, Chile

**Keywords:** Radical prostatectomy, Prostate cancer, Intensity-modulated radiotherapy

## Abstract

**Background:**

The objective of this work was to assess the overall survival, cause-specific survival and biochemical failure-free survival of a contemporary cohort of patients with localized prostate cancer (PCa) treated with intensity-modulated radiation therapy (IMRT) or radical prostatectomy (RP).

**Methods:**

We did a retrospective cohort study of our institution’s registry of patients undergoing either IMRT or RP between January 1999 and March 2010, and assessed Prostate Specific Antigen (PSA), age at diagnosis, Gleason score, and digital rectal examination. Two groups were separated according to RP or IMRT treatment and these groups were in turn divided into risk groups according to the D’Amico classification. Overall survival (OS), cause-specific survival (CSS), mortality from other causes (MOC), and biochemical disease-free survival (BDFS) were assessed.

**Results:**

Twelve-hundred patients were included: 993 in the RP group and 207 in the IMRT group.

The IMRT group had older age, PSA at diagnosis and a significantly higher percentage of cancer on the needle biopsy (p <0.001). Of the 207 patients who underwent IMRT, 54% presented comorbidities. Median follow-up was 91.7 months for the RP group and 76 months for the IMRT group. The OS at 5 and 7 was 96.2, and 93.7 for the RP group respectively and 88.4, and 83.1 for the IMRT group respectively (p <0.001). There were no significant differences in the CSS in relation to treatment received among the low- and high-risk groups, while in the intermediate-risk group, patients who underwent to RP had a higher CSS than patients who underwent IMRT (99.6% vs 94.1%, p = 0.003). The IMRT group had a significantly better BDFS than the RP group (86.4% vs. 74.3%, respectively, p = 0.016).

**Conclusions:**

Patients treated with RP were significantly younger and had a better prognosis than patients treated using IMRT, and according to our results, RP had better outcomes in terms of OS while IMRT had greater MOC. Treatment modality did not affect the CSS.

## Background

Prostate cancer (PCa) is the most common cancer in men. The annual incidence of PCa in the USA is estimated to be 241.740, representing 29% of all cancers [1]. In Chile, PCa is the second leading cause of cancer death in men, with a mortality rate of 20.2/100.000, surpassing lung cancer and only being surpassed by gastric cancer. It is the eighth leading cause of overall death in men [2-4].

Currently, the treatments of choice for low-, intermediate- and high-risk localized PCa are radical prostatectomy (RP) and radiotherapy (RT), which have been considered as curative treatments with comparable oncologic outcomes [5-7]. Because of this, there has been no final consensus as to which treatment is the best choice for a patient with localized PCa. Both RP and RT decrease cause-specific mortality from PCa compared with watchful waiting, as shown in a recent systematic review by Chou et al. [8]. Other systematic reviews [9] show better results in recurrence-free survival at 5 years for RP compared with external RT (14% vs. 39%, respectively, p = 0.04); however, both reviews conclude that the evaluation of treatments is difficult because of limitations in the evidence.

As for RP, surgical techniques have been added, such as the preservation of the neurovascular bundles and laparoscopy or robotic surgery to improve outcomes and decrease complications. Radiotherapy has advanced towards intensity-modulated radiation therapy (IMRT). IMRT is a new technique that modulates the radiation beam so that it achieves a more conformal dose distribution to the target and spare more normal tissues Thus, in PCa, IMRT allows an increase of the prostate radiation dose and reduces toxicity in the rectum and bladder. Sheets et al. [10] showed that increasing the radiation dose improves recurrence-free survival and furthermore concluded that IMRT had fewer gastrointestinal side effects and lower rates of hip fracture than conventional RT. There are several inconclusive systematic reviews comparing radiation treatments [11,12]. However, according to a recent review by Bauman et al. [13], which included 11 studies with over 4500 patients, IMRT would have an advantage over three-dimensional conformal RT (3DCRT) in localized PCa requiring more than 70 Gy, and therefore should become the treatment of choice for these patients. Thus, 3DCRT has largely been supplanted by IMRT as the external beam radiation technique of choice. In 2000, 0.15% of treatments were carried out using IMRT compared with 95.9% in 2008 [10].

To our knowledge, there is only one study comparing IMRT and RP [14], since most studies compare RT (without specifying the RT technique) with surgery. Therefore, our objective was to assess outcomes such as overall survival (OS), cause-specific survival (CSS), mortality from other causes (MOC) and biochemical disease-free survival (BDFS) in a contemporary cohort of over one thousand patients with localized PCa treated with IMRT or RP in a Chilean population.

## Methods

### Patients

We did a retrospective cohort study of the Urology and Radiotherapy Department records of the Pontificia Universidad Católica de Chile for patients with a clinically localized PCa diagnosis who were treated with definitive RP or IMRT (without hormone therapy) between January 1999 and March 2010.

In compliance with the Helsinki Declaration, ethical approval, as well as permission to access the database used in this study, was provided by the local research ethics committee of the Pontificia Universidad Católica de Chile. Due to the retrospective, non interventional nature of this study, no consent was requested by the local research ethics committee.

### Staging and risk groups

Characteristics were assessed prior to treatment, and included age at diagnosis, PSA at diagnosis, Gleason score, percentage of cancer on the needle biopsy (calculated as the sum of the length of PCa in positive cores, divided by the total sum of all core lengths) and digital rectal examination (DRE). A bone and CT scan of the abdomen and pelvis were requested in patients of intermediate and high risk, meeting the NCCN criteria [15]. Patients were stratified according to the D’Amico classification [16] and according to the Charlson comorbidity index (CCI) [17] for the IMRT group (no information was available for the RP group).

### Treatments

Following an explanation of therapeutic alternatives, treatment selection between RP or IMRT was decided by the patient with the urologist. Poor surgery candidates and those who preferred IMRT to PR were referred to radiotherapy.

Patients undergoing surgery underwent open or laparoscopic RP surgery, and lymphadenectomy was performed on patients of high and intermediate risk. For the IMRT technique, a bracing system was used with the patient in the supine position immobilized with a VacLoc (Civco, Iowa) device. Patients underwent CT simulation with a full bladder and empty rectum using an axial slice separation of 3 mm. The treatment volume was defined to include the prostate (for low risk) and prostate plus seminal vesicles (SSVV) for intermediate and high-risk patients, with a safety margin to account for positioning errors and prostate movements (Planning Target Volume (PTV) [18] for 0.8 cm anterior, lateral and superior-inferior to the prostate, and 0.6 cm towards the posterior. PTV for SSVV was 1.2 cm anterior; the other dimensions had the same margins as the prostate PTV). The bladder, femoral heads, rectum, and midrectum (defined as the rectum at the height of the PTV) were also drawn. The dose per fraction was 2 Gy. Initially, 46 Gy were administered to the prostate and seminal vesicles, followed by a 30 Gy boost to the prostate only to complete 76 Gy in 39 fractions over 7 ½ weeks. A 95% coverage of the prescribed dose was required for 95% of the volume. The rectal dose constraints were 77 Gy to <10%, 60 Gy to <30%, and 40 Gy to <60%; the midrectum constraints were 39 Gy to <50%, bladder constraint was 40 Gy to <60%, and the femoral head constraint was 36 Gy to <30%.

### Follow-up

Follow-up time started from the day of surgery in the RP group or from the start of radiotherapy for the IMRT group. Patients were followed up with clinical controls (PSA and DRE) every 3 months for the first 2 years, then every 6 months until 5 years, and then annually.

Biochemical failure was defined as two consecutive PSA findings of 0.2 ng/mL for the RP group and according to the Phoenix consensus (a rise in PSA is greater than or equal to 2 ng/mL above the lower limit reached by the PSA) for IMRT [19].

### Statistical analysis

Death from prostate cancer was considered for all the death certificates that mentioned prostate cancer within the causes of death.

The OS, CSS, MOC and BDFS were analyzed globally and according to D’Amico risk for each group. The Kaplan-Meier method was used and log rank to compare curves. We used a Cox proportional hazard regression model for multivariate analysis. Analysis was performed using IBM SPSS v19.

## Results

### Patient characteristics

A total of 1200 patients were included: 993 in the RP group and 207 in the IMRT group. Patient characteristics are given in Table 1. It was observed that the RP group was significantly younger (p = 0.001) than the IMRT group, with average ages of 63 and 70 years, respectively (CI, 62.6–63.5 and 69–71 years, respectively). Furthermore, patients undergoing IMRT had a significantly higher PSA than those of the RP group, with an average of 9.8 ng/mL for the RP group and 13.6 ng/mL for the IMRT group (p <0.001). The amount of cancer on the needle biopsy was higher in the IMRT group (25.7%) than in the RP group (17.6%) (p <0.001).

**Table 1:** Patient characteristics of the RP and IMRT groups

	**RP**	**IMRT**	**p**
**No. of patients**	993	207	
**Mean age (95% CI)**	63 (62.6–63.5)	70 (69–71)	p < 0.001*
**Mean PSA (95% CI)**	9.8 (9.1–10.5)	13.6 (11.8–16.6)	p < 0.001*
**Mean % PCa biopsy (95% CI)**	17.6 (16.1–18.9)	25.7 (22–29.6)	p < 0.001*
**DRE**			p < 0.001†
**T1 (n/%)**	537/ 53.7%	84/ 40.6%	
**T2 (n/%)**	175/ 18%	70/ 34%	
**T3 (n/%)**	29/ 4%	53/ 26%	
**T4 (n/%)**	0	0	
**Unknown (n/%)**	266/26.7%	0	
**D’Amico classification**			p < 0.001†
**Low risk (n/%)**	194/19.5%	40/19.3%	
**Intermediate risk (n/%)**	525/52.9%	79/37.7	
**High risk (n/%)**	216/21.7%	78/38%	
**Unknown (n/%)**	56/5.6%	10/4.89	

*p values from unpareid t test for two means. **†**p values from chi-square test.

When dividing by risk groups according to D’Amico classification, it was observed that about 70% of patients undergoing RP had low or intermediate risk, while the best percentage of patients undergoing IMRT were of intermediate or high risk, making it a significant difference (p <0.001).

No patients in the RP group received neo-adjuvant Androgen Deprivation therapy (ADT). On the other hand, patients treated with IMRT had neo-adjuvant ADT: in 12%, 34% and 76% for the low, intermediate and high risk group. Of the 87 patients who received ADT, 8 received only peripheral anti-androgen therapy and 79 received central ADT with GnRH analogues. In the high risk 24% was treated with prolonged ADT (≥ 2 years), 39% was treated with short ADT (≤6 years), in 37% the duration of ADT is unknown.

During the study period no adjuvant RT or ADT was indicated after RP. Patients were followed with PSA and if biochemical failure was diagnosed, early RT salvage was indicated. Of 993 patients of the RP group, 253 patients (25%) had biochemical failure, of whom 48 patients received salvage RT with curative intent in our center.

Of the 207 patients who uderwent IMRT, 54% presented comorbidities, the most common being Diabetes Mellitus 2, Coronary Heart Disease and Chronic Obstructive Pulmonary Disease. Over 45% of patients undergoing IMRT had CCI ≥ 1 and 92% had CCI age-adjusted ≥ 1. CCI could not be calculated in the RP group because the information needed was not available.

### Treatment outcomes

**Figure 1:**
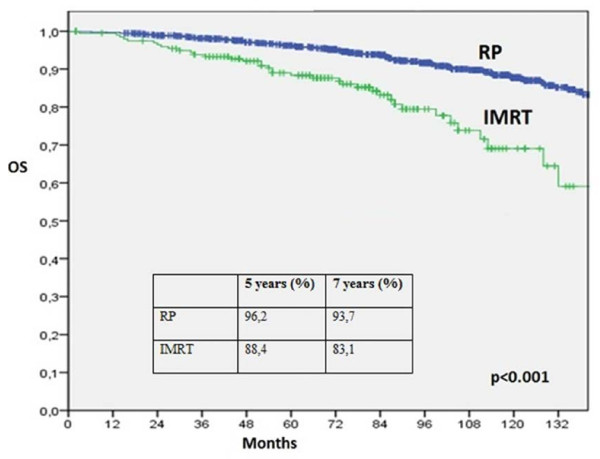
**Overall survival according to treatment.** IMRT: Intensity-Modulated Radiation Therapy. RP: Radical Prostatectomy. OS: Overall Survival.

Median follow-up was 91.7 months for the RP group and 76 months for the IMRT group. OS at 5 and 7 years was 96.2% and 93,7% for the RP group and 88,4% and 83,1% for IMRT group (p <0.001) (Figure 1). Intermediate- and high-risk patients who underwent RP had a longer survival than those who underwent IMRT at 5 and 7 years (p <0.001 to intermediate risk and p = 0.02 to high risk, Table 2).

**Table 2:** Overall survival according to treatment and risk groups to 5 and 7 years

**Overall survival**		**5 years (%)**	**95% CI**	**7 years (%)**	**95% CI**	**p value**
**All patients**	RP	96,2	0.948-0.972	93,7	0.917-0.952	<0.001
	IMRT	88,4	0.827-0.923	83,1	0.760-0.883	
**Low risk**	RP	96,5	0.924-0,984	95,7	0.912-0.979	0.97
	IMRT	97,4	0.825-0.996	97,4	0.825-0.996	
**Intermediate risk**	RP	97,3	0.955-0.984	95,5	0.931-0.971	<0.001
	IMRT	86,3	0,761-0.924	80,4	0.683-0.883	
**High risk**	RP	92,7	0.879-0.956	87,5	0.816-0.916	0.02
	IMRT	85,1	0.729-0.921	77,3	0.625-0.868	

**Figure 2:**
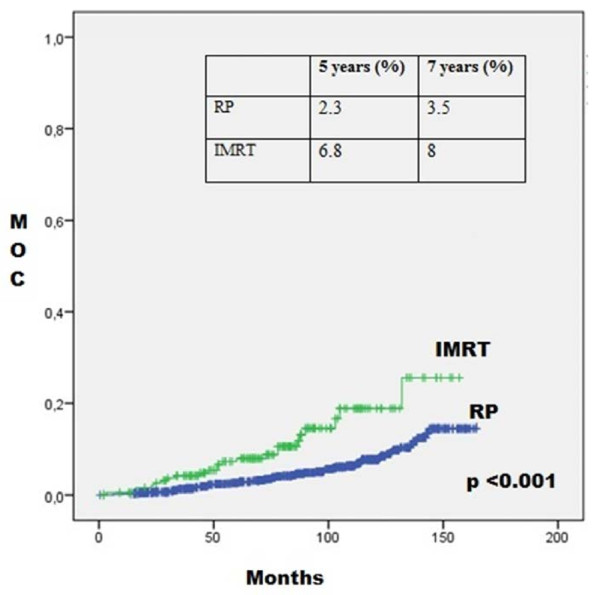
**Mortality from other causes according to treatment.** IMRT: Intensity-Modulated Radiation Therapy. RP: Radical Prostatectomy. MOC: Mortality for other causes.

**Figure 3:**
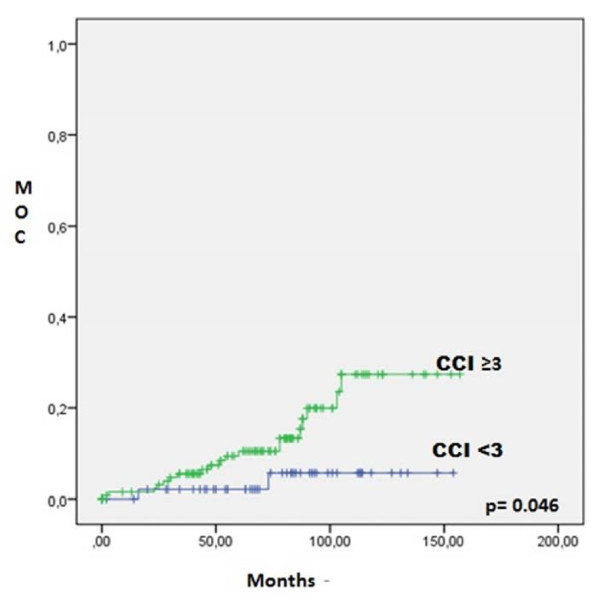
**Mortality from other causes in IMRT group according CCI adjusted for age.** IMRT: Intensity-Modulated Radiation Therapy. MOC: Mortality for other causes. CCI: Charlson Comorbidity Index.

Figure 2 shows MOC for the RP group and IMRT group, which shows that the MOC is higher for the IMRT group at 5 and 7 years (p <0.001). Figure 3 shows MOC for the IMRT group according CCI adjusted for age (categorized into <3 or ≥ 3 points). As expected, patients with greater CCI have higher mortality from other causes (p = 0.046).

**Figure 4:**
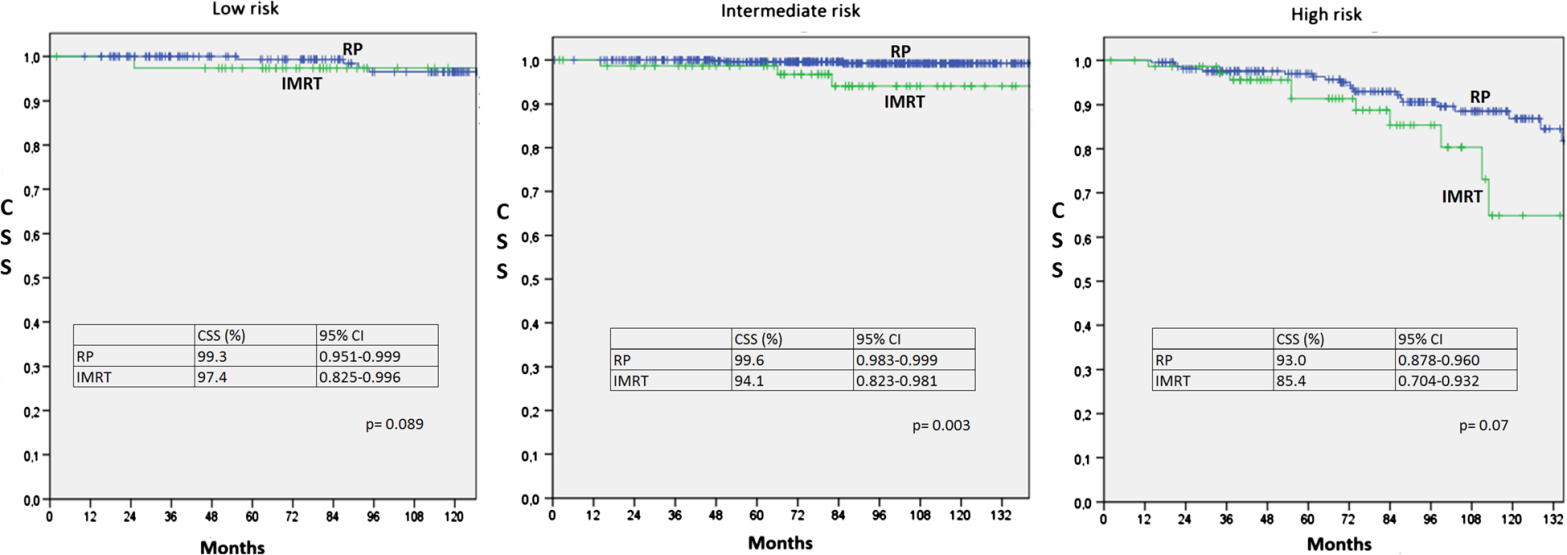
**Cancer-Specific Survival to 7 years according to risk groups.** CI: Confidence Interval. IMRT: Intensity-Modulated Radiation Therapy. RP: Radical Prostatectomy. CSS: Cause-Specific Survival.

Analysis of CSS are shown in Table 3 and Figure 4. We found that CSS is higher in the RP group than IMRT group (98.1 vs 92.1, p < 0.001) and highlighting that there was no significant difference in CSS in the low- and high-risk groups, while in the intermediate-risk group, patients who underwent RP had a statistical significant, although minimal longer cancer-free survival than patients who underwent IMRT at 7 years (99.8% vs 98.6% p = 0.003).

**Table 3:** Cancer-Specific Survival according to treatment and risk groups to 7 years

**Cancer-specific survival**		**7 years (%)**	**95% CI**	**p value**
**All patients**	RP	98.1	0.968-0.989	<0.001
	IMRT	92.1	0.858-0.957	
**Low risk**	RP	99.3	0.951-0.999	0.089
	IMRT	97.4	0.825-0.996	
**Intermediate risk**	RP	99.6	0.983-0.999	0.003
	IMRT	94.1	0.823-0.981	
**High risk**	RP	93.0	0.878-0.960	0.07
	IMRT	85.4	0.704-0.932	

**Figure 5:**
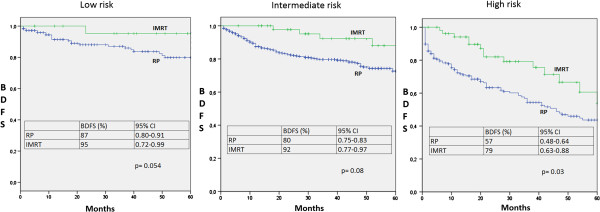
**Biochemical Disease-Free Survival at 36 months according to risk groups.** CI: Confidence Interval. IMRT: Intensity-Modulated Radiation Therapy. RP: Radical Prostatectomy. BDFS: Biochemical Disease-Free Survival.

BDFS was 75% for the RP group and 88% for the IMRT group at 36 months (p = 0.016). The BDFS at 36 months for RP group was 87%, 80% and 56%, for the low-, intermediate- and high-risk groups, respectively .for to the IMRT group, BDFS was 95%, 92% and 79% for the low-, intermediate- and high-risk (Figure 5). Only in the high-risk group there was significant difference in BDFS (p = 0.03).

Multivariate analysis for Cause-Specific Mortality (CSM) (Table 4) showed that Gleason score ≥8, DRE ≥ T2b and DRE ≥ T3 predicted CSM (HR 7.18, HR 5.67 and HR 8.38, respectively). The treatment does not affect the CSM (HR 1.706, 95% CI 0.730–3.987, p = 0.218)

**Table 4:** Multivariate analysis of cancer-specific mortality

**Variable**	**HR**	**95% CI**	**p**
**Age**	0.987*	0.942-1.033	0.567
**RP**			Reference
**IMRT**	1.706	0.730-3.987	0.218
**DRE T1-T2a**			Reference
**DRE T2b-T2c**	5.674	2.205-14.599	0.001
**DRE ≥ T3**	8.380	3.179-22.091	0.001
**Gleason score ≤6**			Reference
**Gleason score =7**	1.397	0.512-3.810	0.514
**Gleason score ≥8**	7.183	2.777-18.574	0.001
**PSA ≤ 10 ng/mL**			Reference
**PSA > 10 and ≤20 ng/mL**	0.333	0.111-0.994	0.049
**PSA > 20 ng/mL**	0.984	0.427-2.268	0.971

*HR for age expressed per year increase.

## Discussion

To our knowledge, this is the first study in Chile and the second in the wider literature comparing IMRT results with RP in a contemporary cohort of patients. The main strength of our study lies in the number of patients and the follow-up of patients, with a median follow-up time of 91 months for the RP group and 76 months for the IMRT group, which provides not only results as BDFS, but also clinical outcomes such as OS and, more important, Cancer-Specific Survival.

The prevalence of surgical treatment over IMRT is highlighted in the studied period where 82.7% of patients received RP versus only 17.3% who were treated with IMRT. This treatment pattern significantly differs from the literature, as for example, the Abdollah study [20], which describes a study of 68,665 patients in the SEER database, of which 67% received radiotherapy. Other studies, such as Schymura et al. [21], with 3500 patients, showed that 31% received RT, 39% RP and 28% did not receive locoregional treatment. Our center began using IMRT routinely in 2001, when only 0.9% of RT treatments in the U.S.A were using this technique [22].

The RP and IMRT groups differed in the baseline characteristics, both in age and other variables (PSA,% of PCa in the biopsy, DRE). Patients who underwent IMRT were older and had a higher risk PCa, which is repeated in different series. For example, in 2009, Aizer et al. [14] compared IMRT with RP and observed that the patients who underwent RT were significantly older and had a higher Gleason score (p <0.001).

Another aspect highlighted in the literature is that patients undergoing RT have higher comorbidity (Charlson comorbidity index) than RP patients. [20]. In the current study, we observed that in the IMRT group, patients with higher age-adjusted CCI had increased mortality from other causes (p = 0.039). The comorbidity of our surgical patients was not systematically studied during this period, although it is likely that those patients with more comorbidities were referred to radiotherapy as described in a previous study [23]. RT treatment was preferred for older patients and those with higher surgical risk because as mentioned, there is no single decisive criterion between RT or RP; and therefore it depends on patient features and preference.

Our results showed a better OS at 5 and 7 years for the RP group than the IMRT group. However, mortality from other causes is higher in the IMRT group and no difference in cancer-specific mortality between the two groups was found. This could be explained by older age and the high comorbidities in the IMRT group.

The best results in terms of OS, despite the higher rate of biochemical failure in the RP group, raise the possibility that a significant percentage of these patients with biochemical recurrence may have received some form of salvage therapy (possibly with RT) while primarily irradiated patients would not have the opportunity to receive a curative salvage treatment like RP in our facilities.

Univariate analysis showed that RP group has a higher CSS than IMRT group. However, when analyzing by risk groups we found only minimal difference in the intermediate-risk group in favor of the RP treatment. Also, the multivariate analysis showed no significant differences between RP and IMRT for CSS.

Regarding BDFS, there were significant global and specific differences between IMRT and RP in the high-risk group. This result is similar to the one presented by Aizer et al. [14]: regarding IMRT or RP treatment, low- and intermediate-risk groups showed no difference in biochemical failure; however, the high-risk group showed a difference in favor of IMRT over RP (62.2% vs. 38.4% respectively, p <0.001), which the authors attributed to the use of hormone therapy in the IMRT group. In our results, it is striking that despite having better BDFS, the IMRT group had worse OS. This could be explained because IMRT patients are typically older and probably have greater comorbidity, as previously discussed, so their OS decreases because of other causes of death (Figure 2).

Currently, IMRT is a therapy recommended by the NCCN [20] that has shown good results in Chile [23], and we know that there has been progress in its application, especially when combined with hormone therapy. The aforementioned study by Aizer et al. [14] concluded that high risk patients who underwent IMRT plus hormone therapy had better BDFS. Meanwhile, a study by Parikh et al. [24] showed a similar result: external RT (method was not specified) combined with ADT was better than surgery plus adjuvant RT for high-risk PCa. We can then assume that the IMRT outcomes in our study could be improved by long term ADT in high risk patients, a protocol that is already being used in our Cancer Center. Currently, there is abundant evidence confirming that patients with high-risk PCa would benefit from prolonged ADT, improving their OS and CSS [25-27]. Another aspect that could benefit the IMRT outcomes is to treat the pelvic nodes that could de potentiality involved in high-risk patients. Prophylactic pelvic RT is recommended by some groups [28] despite not having adequate randomized studies that support it.

A major limitation of our current study is that we do not have the CCI for RP group, which could have allowed us to better clarify the specific causes of mortality in patients who underwent RP. Another limitation is that we have limited information regarding adjuvant therapy (ADT or RT) received by RP group, since we cannot rule out that they have been treated in other centers. Different definitions of biochemical failure may favor the IMRT because the definition of 0.2 ng/mL for recurrence in the RP group is more sensitive to the definition of Phoenix consensus [19] used for the IMRT group. However these definitions are commonly used in the clinic and are crucial to define further treatment promptly; therefore we decided to use them despite these limitations. Finally, retrospective nature of the study is also a weakness because it does not rule out confounding variables that could alter the results. The high percentage of surgery patients suggests a selection bias against IMRT patients and although this bias could be minimized by multivariate analysis it cannot be ruled out.

This study confirms the results of other retrospective studies and emphasizes the importance of randomized trials that minimize selection bias of patients.

We look forward to the results of the study Protect T trial (NCT00632983). This study aims to compare the results of RP, conformal radiotherapy and active surveillance, outcomes include disease-specific survival at 10 years, treatment complications and quality of life. These results are expected in 2016 and have the potential to change the way we face this disease.

## Conclusions

In conclusion, we found that patients treated with RP were significantly younger and had a better prognosis than patients treated with IMRT. RP had better outcomes in terms of OS, however IMRT has greater MOC. There is a need for further prospective studies of both treatments modalities, with a larger number of patients and longer follow-up.

## Abbreviations

PCa: Prostate cancer; PSA: Prostatic specific antigen; CI: Confidence interval; IMRT: Intensity-modulated radiation therapy; RP: Radical prostatectomy; RT: Radiotherapy; ADT: Androgen deprivation therapy; OS: Overall survival; MOC: Mortality for other causes; CSS: Cause-specific survival; BDFS: Biochemical disease-free survival; CCI: Charlson comorbidity index; 3DCRT: Three-dimensional RT; DRE: Digital rectal examination; SSVV: Seminal vesicles; PTV: Planning target volume; HR: Hazard ratio.

## Competing interests

The authors declare that they have no competing interests.

## Authors’ contributions

TM and IFS designed the study; TM, IFS, PB and AZ participated in clinical data collection; TM participated in the statistical analysis; PR drafted and supervised the manuscript. PFB, IFS and TM reviewed the manuscript. All authors read and approved the final manuscript.

## Pre-publication history

The pre-publication history for this paper can be accessed here:

http://www.biomedcentral.com/1471-2407/13/530/prepub
